# (*E*)-Methyl *N*′-(2,4,5-trimeth­oxy­benzyl­idene)hydrazinecarboxyl­ate

**DOI:** 10.1107/S1600536811026705

**Published:** 2011-07-13

**Authors:** Lu-Ping Lv, Tie-Ming Yu, Wen-Bo Yu, Xian-Chao Hu

**Affiliations:** a Department of Chemical Engineering, Hangzhou Vocational and Technical College, Hangzhou 310018, People’s Republic of China; b Research Center of Analysis and Measurement, Zhejiang University of Technology, Hangzhou 310014, People’s Republic of China

## Abstract

The title mol­ecule, C_12_H_16_N_2_O_5_, adopts a *trans* configuration with respect to the C=N bond. In the crystal, inter­molecular N—H⋯O hydrogen bonds link the mol­ecules into chains in [001], and weak inter­molecular C—H⋯O inter­actions further link the chains into corrugated layers parallel to the *bc* plane.

## Related literature

For applications of benzaldehyde­hydrazone derivatives, see: Parashar *et al.* (1988 ▶); Hadjoudis *et al.* (1987 ▶); Borg *et al.* (1999 ▶). For a related structure, see: Shang *et al.* (2007 ▶).
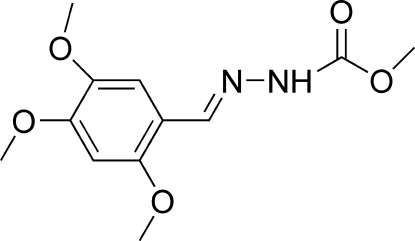


## Experimental

### 

#### Crystal data

C_12_H_16_N_2_O_5_
*M* *_r_* = 268.27
Monoclinic, 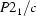

*a* = 9.9897 (15) Å
*b* = 17.606 (3) Å
*c* = 8.0801 (12) Å
β = 111.806 (4)°
*V* = 1319.4 (3) Å^3^
*Z* = 4
Mo *K*α radiation
μ = 0.11 mm^−1^
*T* = 223 K
0.18 × 0.15 × 0.12 mm

#### Data collection

Bruker SMART CCD area-detector diffractometer
Absorption correction: multi-scan (*SADABS*; Bruker, 2002 ▶) *T*_min_ = 0.971, *T*_max_ = 0.979
9747 measured reflections
2318 independent reflections
1836 reflections with *I* > 2σ(*I*)
*R*_int_ = 0.039

#### Refinement

*R*[*F* ^2^ > 2σ(*F* ^2^)] = 0.040
*wR*(*F* ^2^) = 0.112
*S* = 1.10
2318 reflections
173 parameters
H-atom parameters constrained
Δρ_max_ = 0.19 e Å^−3^
Δρ_min_ = −0.19 e Å^−3^

### 

Data collection: *SMART* (Bruker, 2002 ▶); cell refinement: *SAINT* (Bruker, 2002 ▶); data reduction: *SAINT*; program(s) used to solve structure: *SHELXS97* (Sheldrick, 2008 ▶); program(s) used to refine structure: *SHELXL97* (Sheldrick, 2008 ▶); molecular graphics: *SHELXTL* (Sheldrick, 2008 ▶); software used to prepare material for publication: *SHELXTL*.

## Supplementary Material

Crystal structure: contains datablock(s) I, global. DOI: 10.1107/S1600536811026705/cv5126sup1.cif

Structure factors: contains datablock(s) I. DOI: 10.1107/S1600536811026705/cv5126Isup2.hkl

Supplementary material file. DOI: 10.1107/S1600536811026705/cv5126Isup3.cml

Additional supplementary materials:  crystallographic information; 3D view; checkCIF report

## Figures and Tables

**Table 1:** Hydrogen-bond geometry (Å, °)

*D*—H⋯*A*	*D*—H	H⋯*A*	*D*⋯*A*	*D*—H⋯*A*
N2—H2⋯O4^i^	0.86	2.09	2.8403 (18)	146
C8—H8*A*⋯O3^ii^	0.96	2.56	3.423 (2)	150

Symmetry codes: (i) 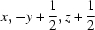; (ii) 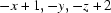.

**Fig. 1.**
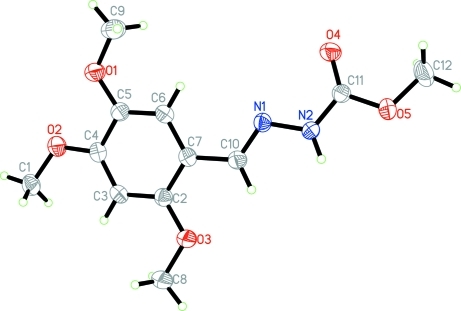
Molecular structure of (I), showing 40% probability displacement ellipsoids and the atomic numbering.

## supplementary crystallographic information

### Comment

Benzaldehydehydrazone derivatives have received considerable attentions due to their pharmacological activity (Parashar *et al.*, 1988) and photochromic properties (Hadjoudis *et al.*, 1987). They also serve as intermidiates of 1,3,4-oxadiazoles, which have been reported to be versatile compounds with many properties (Borg *et al.*, 1999). As a further investigation of this type of compounds, we report herein the crystal structure of the title compound (I).

The title compound (Fig.1)  adopts a trans configuration with respect to the C═N bond. The hydrazine carboxylic acid methyl ester group is slightly twisted away from the attached ring. The dihedral angle between the benzene ring and the C10/C11/C12//N1/N2/O4/O5 plane is 14.23 (5)°. The bond lengths and angles agree with those observed for (E)-methyl N'-(4-hydroxybenzylidene)hydrazinecarboxylate (Shang *et al.*, 2007).

In the crystal structure, intermolecular N—H···O hydrogen bonds (Table 1) link molecules into chains in [001], and weak intermolecular C—H···O interactions (Table 1) link further these chains into corrugated layers parallel to *bc* plane.

### Experimental

2,4,5-Trimethoxybenzaldehyde (1.96g, 0.01mol) and methyl hydrazinecarboxylate (0.9g, 0.01mol) were dissolved in stirred methanol (20ml) and left for 5.2h at room temperature. The resulting solid was filtered off and recrystallized from ethanol to give the title compound in 91% yield.  Crystals suitable for X-ray analysis were obtained by slow evaporation of a ethanol solution at room temperature (m.p. 432-435 K).

### Refinement

H atoms were geometrically positioned (C—H  0.93 - 0.96 Å; N—H 0.86 Å),  and refined using a riding model, with U_iso_(H) = 1.2-1.5 U_eq_(C, N).

### Crystal data

**Table:**

C_12_H_16_N_2_O_5_	*F*(000) = 568
*M**_r_* = 268.27	*D*_x_ = 1.350 Mg m^−^^3^
Monoclinic, *P*2_1_/*c*	Mo *K*α radiation, λ = 0.71073 Å
Hall symbol: -P 2ybc	Cell parameters from 2318 reflections
*a* = 9.9897 (15) Å	θ = 2.2–25.0°
*b* = 17.606 (3) Å	µ = 0.11 mm^−^^1^
*c* = 8.0801 (12) Å	*T* = 223 K
β = 111.806 (4)°	Block, colourless
*V* = 1319.4 (3) Å^3^	0.18 × 0.15 × 0.12 mm
*Z* = 4	

### Data collection

**Table:**

Bruker SMART CCD area-detector diffractometer	2318 independent reflections
Radiation source: fine-focus sealed tube	1836 reflections with *I* > 2σ(*I*)
graphite	*R*_int_ = 0.039
φ and ω scans	θ_max_ = 25.0°, θ_min_ = 2.2°
Absorption correction: multi-scan (*SADABS*; Bruker, 2002)	*h* = −11→11
*T*_min_ = 0.971, *T*_max_ = 0.979	*k* = −20→20
9747 measured reflections	*l* = −9→9

### Refinement

**Table:**

Refinement on *F*^2^	Secondary atom site location: difference Fourier map
Least-squares matrix: full	Hydrogen site location: inferred from neighbouring sites
*R*[*F*^2^ > 2σ(*F*^2^)] = 0.040	H-atom parameters constrained
*wR*(*F*^2^) = 0.112	*w* = 1/[σ^2^(*F*_o_^2^) + (0.0529*P*)^2^ + 0.1933*P*] where *P* = (*F*_o_^2^ + 2*F*_c_^2^)/3
*S* = 1.10	(Δ/σ)_max_ < 0.001
2318 reflections	Δρ_max_ = 0.19 e Å^−^^3^
173 parameters	Δρ_min_ = −0.19 e Å^−^^3^
0 restraints	Extinction correction: *SHELXL97* (Sheldrick, 2008), Fc^*^=kFc[1+0.001xFc^2^λ^3^/sin(2θ)]^-1/4^
Primary atom site location: structure-invariant direct methods	Extinction coefficient: 0.014 (2)

### Special details

**Table:**

Geometry. All esds (except the esd in the dihedral angle between two l.s. planes) are estimated using the full covariance matrix. The cell esds are taken into account individually in the estimation of esds in distances, angles and torsion angles; correlations between esds in cell parameters are only used when they are defined by crystal symmetry. An approximate (isotropic) treatment of cell esds is used for estimating esds involving l.s. planes.
Refinement. Refinement of F^2^ against ALL reflections. The weighted R-factor wR and goodness of fit S are based on F^2^, conventional R-factors R are based on F, with F set to zero for negative F^2^. The threshold expression of F^2^ > 2sigma(F^2^) is used only for calculating R-factors(gt) etc. and is not relevant to the choice of reflections for refinement. R-factors based on F^2^ are statistically about twice as large as those based on F, and R- factors based on ALL data will be even larger.

### Fractional atomic coordinates and isotropic or equivalent isotropic displacement parameters (Å^2^)

**Table:**

	*x*	*y*	*z*	*U*_iso_*/*U*_eq_	
C1	0.0907 (2)	−0.07569 (10)	0.1659 (3)	0.0495 (5)	
H1A	0.0345	−0.0999	0.0554	0.074*	
H1B	0.0279	−0.0567	0.2218	0.074*	
H1C	0.1565	−0.1118	0.2432	0.074*	
C4	0.25538 (17)	0.02726 (9)	0.2728 (2)	0.0379 (4)	
C5	0.31441 (18)	0.09331 (9)	0.2291 (2)	0.0393 (4)	
C9	0.3154 (3)	0.17706 (12)	−0.0014 (3)	0.0740 (7)	
H9A	0.2793	0.1803	−0.1291	0.111*	
H9B	0.4188	0.1796	0.0445	0.111*	
H9C	0.2780	0.2185	0.0455	0.111*	
C6	0.40772 (18)	0.13694 (9)	0.3631 (2)	0.0375 (4)	
H6	0.4482	0.1800	0.3339	0.045*	
C3	0.28653 (17)	0.00903 (9)	0.4492 (2)	0.0374 (4)	
H3	0.2450	−0.0338	0.4779	0.045*	
C2	0.37954 (17)	0.05417 (9)	0.5844 (2)	0.0366 (4)	
C8	0.3448 (2)	−0.02382 (11)	0.8084 (3)	0.0529 (5)	
H8A	0.3788	−0.0283	0.9358	0.079*	
H8B	0.3666	−0.0696	0.7588	0.079*	
H8C	0.2424	−0.0158	0.7618	0.079*	
C7	0.44350 (17)	0.11824 (9)	0.5432 (2)	0.0349 (4)	
C10	0.54924 (18)	0.16166 (9)	0.6853 (2)	0.0381 (4)	
H10	0.5663	0.1494	0.8034	0.046*	
C11	0.81188 (18)	0.30059 (9)	0.7763 (2)	0.0360 (4)	
C12	1.0210 (2)	0.37093 (12)	0.9307 (3)	0.0608 (6)	
H12A	1.0813	0.3852	1.0498	0.091*	
H12B	0.9823	0.4157	0.8618	0.091*	
H12C	1.0771	0.3431	0.8769	0.091*	
N1	0.61921 (14)	0.21635 (7)	0.65137 (17)	0.0357 (4)	
N2	0.71776 (15)	0.25135 (8)	0.79917 (17)	0.0404 (4)	
H2	0.7189	0.2418	0.9040	0.049*	
O1	0.27162 (15)	0.10777 (7)	0.04985 (16)	0.0559 (4)	
O2	0.16975 (13)	−0.01434 (7)	0.13198 (16)	0.0520 (4)	
O3	0.41409 (14)	0.03895 (7)	0.76186 (16)	0.0501 (4)	
O4	0.81346 (14)	0.32263 (7)	0.63555 (16)	0.0488 (4)	
O5	0.90455 (14)	0.32388 (7)	0.93597 (16)	0.0530 (4)	

### Atomic displacement parameters (Å^2^)

**Table:**

	*U*^11^	*U*^22^	*U*^33^	*U*^12^	*U*^13^	*U*^23^
C1	0.0485 (11)	0.0418 (10)	0.0529 (11)	−0.0116 (8)	0.0126 (9)	−0.0047 (8)
C4	0.0334 (9)	0.0372 (9)	0.0420 (10)	−0.0014 (7)	0.0128 (8)	−0.0043 (7)
C5	0.0406 (10)	0.0415 (9)	0.0363 (10)	−0.0020 (8)	0.0150 (8)	−0.0014 (7)
C9	0.1031 (19)	0.0639 (14)	0.0460 (12)	−0.0276 (13)	0.0170 (12)	0.0066 (10)
C6	0.0379 (9)	0.0362 (9)	0.0411 (10)	−0.0031 (7)	0.0178 (8)	−0.0002 (7)
C3	0.0343 (9)	0.0349 (9)	0.0460 (11)	−0.0018 (7)	0.0183 (8)	0.0003 (7)
C2	0.0339 (9)	0.0380 (9)	0.0391 (10)	0.0016 (7)	0.0150 (7)	0.0016 (7)
C8	0.0610 (13)	0.0518 (11)	0.0482 (11)	−0.0143 (9)	0.0230 (10)	0.0053 (9)
C7	0.0324 (8)	0.0345 (9)	0.0394 (10)	0.0011 (7)	0.0150 (7)	−0.0022 (7)
C10	0.0396 (9)	0.0404 (9)	0.0362 (9)	−0.0017 (8)	0.0164 (8)	−0.0006 (7)
C11	0.0407 (9)	0.0346 (9)	0.0348 (10)	−0.0027 (7)	0.0163 (8)	−0.0030 (7)
C12	0.0441 (11)	0.0646 (13)	0.0714 (15)	−0.0194 (10)	0.0187 (10)	−0.0095 (11)
N1	0.0356 (7)	0.0374 (7)	0.0337 (8)	−0.0032 (6)	0.0124 (6)	−0.0038 (6)
N2	0.0460 (8)	0.0475 (8)	0.0281 (8)	−0.0127 (7)	0.0141 (7)	−0.0033 (6)
O1	0.0700 (9)	0.0561 (8)	0.0372 (7)	−0.0229 (7)	0.0146 (6)	0.0005 (6)
O2	0.0565 (8)	0.0511 (8)	0.0439 (7)	−0.0195 (6)	0.0132 (6)	−0.0063 (6)
O3	0.0594 (8)	0.0510 (8)	0.0386 (7)	−0.0180 (6)	0.0168 (6)	0.0013 (5)
O4	0.0612 (8)	0.0506 (7)	0.0403 (8)	−0.0135 (6)	0.0255 (6)	−0.0020 (6)
O5	0.0510 (8)	0.0632 (8)	0.0408 (7)	−0.0249 (6)	0.0125 (6)	−0.0062 (6)

### Geometric parameters (Å, °)

**Table:**

C1—O2	1.423 (2)	C2—C7	1.396 (2)
C1—H1A	0.9600	C8—O3	1.426 (2)
C1—H1B	0.9600	C8—H8A	0.9600
C1—H1C	0.9600	C8—H8B	0.9600
C4—O2	1.3562 (19)	C8—H8C	0.9600
C4—C3	1.379 (2)	C7—C10	1.455 (2)
C4—C5	1.407 (2)	C10—N1	1.278 (2)
C5—C6	1.372 (2)	C10—H10	0.9300
C5—O1	1.3730 (19)	C11—O4	1.2072 (19)
C9—O1	1.409 (2)	C11—N2	1.341 (2)
C9—H9A	0.9600	C11—O5	1.343 (2)
C9—H9B	0.9600	C12—O5	1.442 (2)
C9—H9C	0.9600	C12—H12A	0.9600
C6—C7	1.403 (2)	C12—H12B	0.9600
C6—H6	0.9300	C12—H12C	0.9600
C3—C2	1.391 (2)	N1—N2	1.3790 (18)
C3—H3	0.9300	N2—H2	0.8600
C2—O3	1.371 (2)		
			
O2—C1—H1A	109.5	O3—C8—H8B	109.5
O2—C1—H1B	109.5	H8A—C8—H8B	109.5
H1A—C1—H1B	109.5	O3—C8—H8C	109.5
O2—C1—H1C	109.5	H8A—C8—H8C	109.5
H1A—C1—H1C	109.5	H8B—C8—H8C	109.5
H1B—C1—H1C	109.5	C2—C7—C6	118.29 (15)
O2—C4—C3	124.82 (15)	C2—C7—C10	119.92 (15)
O2—C4—C5	115.38 (15)	C6—C7—C10	121.72 (15)
C3—C4—C5	119.79 (15)	N1—C10—C7	121.40 (15)
C6—C5—O1	125.66 (15)	N1—C10—H10	119.3
C6—C5—C4	119.35 (15)	C7—C10—H10	119.3
O1—C5—C4	114.99 (14)	O4—C11—N2	126.36 (16)
O1—C9—H9A	109.5	O4—C11—O5	124.16 (15)
O1—C9—H9B	109.5	N2—C11—O5	109.47 (14)
H9A—C9—H9B	109.5	O5—C12—H12A	109.5
O1—C9—H9C	109.5	O5—C12—H12B	109.5
H9A—C9—H9C	109.5	H12A—C12—H12B	109.5
H9B—C9—H9C	109.5	O5—C12—H12C	109.5
C5—C6—C7	121.62 (15)	H12A—C12—H12C	109.5
C5—C6—H6	119.2	H12B—C12—H12C	109.5
C7—C6—H6	119.2	C10—N1—N2	114.97 (13)
C4—C3—C2	120.49 (15)	C11—N2—N1	118.74 (13)
C4—C3—H3	119.8	C11—N2—H2	120.6
C2—C3—H3	119.8	N1—N2—H2	120.6
O3—C2—C3	123.04 (14)	C5—O1—C9	117.49 (14)
O3—C2—C7	116.58 (14)	C4—O2—C1	118.08 (14)
C3—C2—C7	120.38 (15)	C2—O3—C8	117.95 (13)
O3—C8—H8A	109.5	C11—O5—C12	115.01 (14)
			
O2—C4—C5—C6	−176.97 (14)	C5—C6—C7—C10	176.10 (15)
C3—C4—C5—C6	3.0 (2)	C2—C7—C10—N1	173.58 (15)
O2—C4—C5—O1	2.7 (2)	C6—C7—C10—N1	−3.6 (2)
C3—C4—C5—O1	−177.36 (15)	C7—C10—N1—N2	−178.78 (14)
O1—C5—C6—C7	178.98 (15)	O4—C11—N2—N1	6.0 (3)
C4—C5—C6—C7	−1.4 (3)	O5—C11—N2—N1	−175.46 (13)
O2—C4—C3—C2	177.90 (15)	C10—N1—N2—C11	169.23 (15)
C5—C4—C3—C2	−2.0 (2)	C6—C5—O1—C9	−6.7 (3)
C4—C3—C2—O3	179.82 (14)	C4—C5—O1—C9	173.66 (17)
C4—C3—C2—C7	−0.5 (2)	C3—C4—O2—C1	8.7 (2)
O3—C2—C7—C6	−178.24 (14)	C5—C4—O2—C1	−171.32 (15)
C3—C2—C7—C6	2.1 (2)	C3—C2—O3—C8	−3.5 (2)
O3—C2—C7—C10	4.5 (2)	C7—C2—O3—C8	176.82 (15)
C3—C2—C7—C10	−175.19 (14)	O4—C11—O5—C12	−7.7 (2)
C5—C6—C7—C2	−1.1 (2)	N2—C11—O5—C12	173.73 (15)

### Hydrogen-bond geometry (Å, °)

**Table:**

*D*—H···*A*	*D*—H	H···*A*	*D*···*A*	*D*—H···*A*
N2—H2···O4^i^	0.86	2.09	2.8403 (18)	146
C8—H8A···O3^ii^	0.96	2.56	3.423 (2)	150

Symmetry codes: (i) *x*, −*y*+1/2, *z*+1/2; (ii) −*x*+1, −*y*, −*z*+2.
